# Draft Genome Sequence of Alkalicoccus halolimnae BZ-SZ-XJ29^T^, a Moderately Halophilic Bacterium Isolated from a Salt Lake

**DOI:** 10.1128/MRA.00500-20

**Published:** 2020-07-02

**Authors:** Yingjie Zhang, Dacheng Qiu, Ziya Liao, Baisuo Zhao

**Affiliations:** a Graduate School, Chinese Academy of Agricultural Sciences, Beijing, People’s Republic of China; Portland State University

## Abstract

The moderate halophile Alkalicoccus halolimnae BZ-SZ-XJ29^T^ grows optimally in a relative broad range of 8.3% to 12.3% (wt/vol) NaCl. The draft genome consists of approximately 3.66 Mb and contains 3,534 putative genes. Various genes involved in osmotic stress were predicted, providing pertinent insights into specific adaptations to the hypersaline environment.

## ANNOUNCEMENT

The mesophilic moderately halophilic bacterium Alkalicoccus halolimnae BZ-SZ-XJ29^T^ was aerobically isolated from a mixture of water and sediment from a salt lake in Xinjiang Uyghur Autonomous Region, China (1). Its growth occurs in the ranges of 4.3% to 24.3% (wt/vol) NaCl, pH 6.0 to 10.5, and 5°C to 41°C. To gain insight into the osmotic adaptive strategies of hypersaline stress, the draft genome of strain BZ-SZ-XJ29^T^ was sequenced using an Illumina HiSeq 4000 platform.

Total genomic DNA (2 μg) was extracted from strain BZ-SZ-XJ29^T^ grown under optimal conditions, as described previously (1), using a microbial DNA isolation kit (iTOP, Beijing, China) following the manufacturer’s instructions. A library for genome sequencing was constructed using the whole-genome shotgun approach with the TruSeq DNA sample preparation kit (Illumina, USA), HiSeq PE cluster kit v4-cBot (Illumina), and HiSeq 3000/4000 SBS kit (Illumina) (2, 3). Sequencing was performed with a paired-end read length of 2 × 150 bp at approximately 200× coverage. The filtered reads were quality trimmed using Quake and the Burrows-Wheeler Aligner (BWA) with the default program parameters and were *de novo* assembled into contigs using SOAPdenovo2 (4). A total of 5,027,473 reads with a total length of 3,668,659 bp were assembled into 59 contigs, with a GC content of 44.9% and an *N*_50_ value of 241,109 bp. Automatic annotation was performed using the NCBI Prokaryotic Genome Annotation Pipeline (PGAP) (https://www.ncbi.nlm.nih.gov/genome/annotation_prok). Subsequently, the genome files were uploaded to the IMG-ER tool (https://img.jgi.doe.gov/cgi-bin/submit/main.cgi) for functional annotation. Among the 3,534 genes identified, 3,435 were potential protein-coding genes. Also predicted were 55 RNAs, including 4 rRNAs (2 5S RNAs, 1 16S RNA, and 1 23S RNA), 45 tRNAs, and 6 other RNAs.

Genome sequence analysis showed the presence of a number of genes encoding putative proteins potentially related to the osmotic strategies for surviving in a hypersaline environment. Identified were one gene cluster (*ectA*, *ectB*, and *ectC*) for ectoine biosynthesis from aspartate semialdehyde, the *betA* gene and *betB* gene for glycine betaine biosynthesis from choline, the *glnA* gene for l-glutamine biosynthesis from l-glutamate, the *proV* gene and *proW* gene for the glycine betaine/proline ABC transporter, the *opuAC* and *opuBD* genes for the osmoprotectant (i.e., choline, glycine betaine, and proline) transport system (ABC transporters), and the *opuD* gene for glycine betaine/proline transporters (betaine/carnitine/choline transporter [BCCT] family). All genes mentioned are important for maintaining osmotic balance though the “compatible solutes strategy” under high-salt conditions. Furthermore, four genes coding for Na^+^/solute symporters (5–8), nine genes coding for the multisubunit Na^+^/H^+^ antiporter (9, 10), and three genes coding for a monovalent cation/proton antiporter (11) were predicted. These genes might be involved in salt stress by maintaining Na^+^ homeostasis. Also detected were four genes (three TrkA type and one TrkH type) responsible for K^+^ uptake systems, implying that strain BZ-SZ-XJ29^T^ may gain rapidly isosmotic cytoplasm though K^+^ as an osmolyte when coping with osmotic shock (12). As described above, many predicted genes in the genome of strain BZ-SZ-XJ29^T^ offer valuable insights to reveal the adaptive mechanisms for maintaining osmotic balance and Na^+^ homeostasis under conditions of elevated salinity.

### Data availability.

The draft genome sequence of Alkalicoccus halolimnae BZ-SZ-XJ29^T^ has been deposited at GenBank under the accession number VPFE00000000. The raw sequencing reads have been submitted to the Sequence Read Archive (SRA accession number SRR9943993) and are available at NCBI under BioProject number PRJNA559242 and BioSample number SAMN12530361.
